# Targeted Gene Silencing BRAF Synergized Photothermal Effect Inhibits Hepatoma Cell Growth Using New GAL-GNR-siBRAF Nanosystem

**DOI:** 10.1186/s11671-020-03340-x

**Published:** 2020-05-24

**Authors:** Yanling Liu, Manman Tan, Yujuan Zhang, Wei Huang, Liangliang Min, Shanshan Peng, Keng Yuan, Li Qiu, Weiping Min

**Affiliations:** 1 Institute of Immunotherapy, Nanchang University, Nanchang, 330006 Jiangxi China; 2 Jiangxi University of Technology, Nanchang, Jiangxi 330098 China; 3 Jiangxi Institute of Medical Sciences Nanchang, Nanchang, 330006 Jiangxi China; 4 Department of Surgery, Pathology and Oncology, University of Western Ontario, London, N6A 5A5 Canada; 5 Department of Endocrinology and Metabolism, Peking University People’s Hospital, Beijing, China

**Keywords:** Gold nanorods, BRAF, siRNA, Liver cancer, Galactose

## Abstract

Liver cancer is one of the most common malignancies worldwide. The RAF kinase inhibitors are effective in the treatment of hepatocellular carcinoma (HCC); therefore, inhibition of the BRAF/MEK/ERK pathway has become a new therapeutic strategy for novel HCC therapy. However, targeted specific delivery systems for tumors are still significant obstacle to clinical applications. Galactose (GAL) can target the asialoglycoprotein receptor (ASGPR) that is highly expressed on liver cancer cells. In this study, we designed a novel multifunctional nanomaterial GAL-GNR-siBRAF which consists of three parts, GAL as the liver cancer-targeting moiety, golden nanorods (GNR) offering photothermal capability under near infrared light, and siRNA specifically silencing BRAF (siBRAF). The nanocarrier GAL-GNR-siBRAF showed high siRNA loading capacity and inhibited the degradation of siRNA in serum. Compared with naked gold nanorods, GAL-GNR-siBRAF possessed lower biotoxicity and higher efficacy of gene silencing. Treatment with GAL-GNR-siBRAF significantly downregulated the expression of BRAF and impaired proliferation, migration, and invasion of liver cancer cells. Moreover, combinatorial photothermal effects and BRAF knockdown by GAL-GNR-siBRAF effectively given rise to tumor cell death. Therefore, our study developed a new type of targeted multi-functional nanomaterial GAL-GNR-siBRAF for the treatment of liver cancer, which provides ideas for the development of new clinical treatment methods.

## Introduction

Hepatocellular carcinoma (HCC) is a major global health problem [1]. It is the sixth most common cancer in the world, and the third leading cause of cancer-related death [2, 3]. Most HCC cases occur in East Asia and sub-Saharan Africa. However, the incidence has been rising in some developed countries, including France, Britain, Japan, and the USA [4]. The standard treatment for early HCC is surgical resection of the tumor. After surgery, the 5-year survival rate can range from 89 to 93% [5]. Unfortunately, only a small percentage of HCC patients (about 20–30%) are diagnosed at an early stage; most HCC patients (> 70%) are found at an advanced scene and cannot undergo surgical resection. Other treatment options include liver transplantation, transcatheter arterial chemoembolization (TACE), and systemic chemotherapy [6]. However, liver transplantation is limited by the supply, and incomplete TACE embolization can lead to treatment failure, with significant side effects of systemic chemotherapy, and poor overall prognosis. Therefore, new treatment methods for hepatocellular carcinoma are highly clinically demanding [7].

The RAS/RAF signaling pathway plays an essential role in the development of liver cancer, and BRAF is one of the essential cancer-associated genes in this pathway. Genetic alterations in these genes often result in two cascaded disorders. Abnormal activation of the RAS/RAF signaling pathway is associated with poor prognosis in cancer patients [8]. BRAF is the most frequently mutated gene in the RAF family, and targeting the RAS/RAF pathway is a novel therapeutic strategy for the treatment of HCC [8–10]. Since the RAF kinase inhibitor sorafenib has been shown to be useful in the treatment of HCC, thus, BRAF mutations have become the preferred target for HCC therapy [8]. More specifically, BRAF mutations have become a desirable target for the treatment of advanced HCC, as the clinical development of the RAF kinase inhibitor sorafenib has been found in HCC treatment in Asia, Europe, and the USA [11]. Compared with placebo, sorafenib increased overall survival prolonging the median overall survival of patients with advanced HCC [12–14]. However, sorafenib has poor tumor-targeting ability, which can lead to adverse side effects such as high blood pressure, hair loss, and nausea [15]. Drugs that target liver cancer with high specificity and block RAF remain to be further explored.

The asialoglycoprotein receptor (ASGPR), also known as the liver galactose receptor, is a C-type lectin expressed on the sinusoidal surface of hepatocytes [16]. ASGPR is considered to be an essential target for liver nanostructures because it plays an important role in binding, internalization, and elimination of substances with terminal galactose residues [17]. Several monosaccharides (galactose, mannose, lactose, N-acetylgalactosamine, and sialic acid) have been reported to interact with ASGPR to varying degrees, and galactose showed higher affinity for ASGPR [18]. Because ASGPR is strongly exposed to the surface of hepatic parenchymal cells [19], the receptor has a strong affinity for galactose and galactosylated prodrugs or delivery systems targeting the liver. Galactose nanoparticles [20], galactose micelles [21], and galactose liposomes [22] have been identified as hepatic targeting drug systems, which target specifically for hepatocellular carcinoid cells.

Gold nanorods (GNR) are rod-shaped gold nanoparticles that have significant advantages as nanocarriers [23]. Gold nanorods (GNR) have excellent biocompatibility and can be used for stable delivery of siRNA [24]; they have a large specific surface area, which allows flexible modification of specific tumor-targeting adaptors [25]; localized surface plasmon resonance (LSPR) has high photothermal conversion efficiency under near-infrared light irradiation and has become an excellent photothermal anti-tumor material [26]. The maximum photothermal efficiency can be achieved by adjusting the aspect ratio of the gold nanorods (GNR). The in vitro and in vivo toxicity of gold nanoparticles depends on their size, surface charge, and surface coating [27]. However, cetyltrimethylammonium bromide (CTAB) is an essential active agent in the synthesis of gold nanorods, which has apparent cytotoxicity and limits biological applications [28].

RNA interference (RNAi) has become a promising approach to cancer treatment because it effectively knocks out or silences target genes by small interfering RNA (siRNA) [29]. Recently, siRNA molecules have entered the human trial phase and are considered to be promising ways to treat multiple mutant gene cancers and tumors [30]. However, the application of siRNA still faces enormous challenges such as serum instability (degradation by nucleases in the extracellular environment) and off-target effects [31]. Moreover, siRNAs are negatively charged, which prevent them from binding to negatively charged cell membranes [32]. Because of these characteristics, siRNA itself is unlikely to be delivered directly to cells. The stability of siRNA can be improved by chemical modification of siRNA or by inserting siRNA loaders into protective carrier materials [33].

In this study, we have newly constructed a multifunctional nanocarrier GAL-GNR-siBRAF. The system uses gold nanorods with optical heat-generating ability as the inner core and externally modified GAL (d-galactose) with specific targeting of liver tumors. This system reduced the biological toxicity of CTAB on the surface of gold nanorods and showed high siRNA loading capacity and can be used to silence BRAF gene in liver cancer effectively. The application of GAL-GNR-siBRAF attenuated proliferation, invasion, and migration of liver cancer cells significantly. In addition, the GAL-GNR-siBRAF simultaneously induced gene silencing of BRAF and photothermal effects which achieved synergistic efficacy in the killing ability of tumor cells, providing a new way of thinking for the development of clinical treatment of liver cancer.

## Material and Methods

### Cell Line

The mouse hepatocellular carcinoma cell line Hepa1-6 was purchased from the Stem Cell Bank, Chinese Academy of Sciences. Cells were cultured in DMEM medium (Life Technologies, Carlsbad, CA) containing 10% FBS at 37 °C with 5% CO_2_.

### Synthesis of BRAF siRNA

The sequence for siRNA targeting BRAF gene is 5′-GCUUACUGGAGAGGAGUUACA-3′ and was synthesized by Dharmacon, Inc. (Lafayette, CO, USA). The commercially available targeted luciferase gene siGL2 was used as a negative control siRNA.

### Synthesis of CTAB-GNR

Water-soluble gold nanorods were synthesized using a seed-mediated growth pathway [34].The seed solution was manufactured as follows: 1 mL of cetyltrimethylammonium bromide (CTAB) (0.2 M) (Sinopharm) was gently mixed with 1 mL of HAuCl_4_ (0.5 mM) (Sinopharm). Stirred the solution at 28 °C, mixed thoroughly and added 0.12 mL cold NaBH_4_ (0.01 M) (Sinopharm) until the obtained seed solution turned brown and remained in reserve. The growth solution was then synthesized as follows: 50 mL of CTAB (0.2 M) and 50 mL of HAuCl_4_ (1 mM), 2.5 mL AgNO_3_ (4 mM) (Sinopharm) were lightly mixed at 28 °C. After thorough mild mixing, 670 μL of ascorbic acid (0.079 M) was added when the color of the solution will change from dark yellow to colorless. One hundred twenty microliters of the seed solution was added under gentle agitation at 28 °C, and the color gradually became transparent from purple to purple-black. After 24 h of stirring at the constant temperature, the solution was centrifuged at 12,000 rpm for 10 min to remove additional CTAB and vacuum freeze-dried into GNR powder for future use.

### Synthesis of MUA-PEI and GAL-PEI-MUA

Mercaptoundecanoic acid (MUA; 654 mg) was dissolve in 30 mL of chloroform (CHCl_3_) and then incubated with100 mmol of 1-ethyl-3-[3-dimethylaminopropyl] carbodiimide hydrochloride (EDC) and 100 mmol of N-hydroxysuccinimide (NHS) for 15 min at room temperature. Next, 100 mmol polyethyleneimine (PEI) was added to the above solution. After 24 h reaction at room temperature, an equal volume of deionized water was used to extract the water-soluble PEI-MUA. The same method was used to complete the activation of d-galactose in aqueous solution. Then, an excess of activated d-galactose (500 mmol) was added to the above water-soluble PEI-MUA solution, and the reaction was sufficiently carried out at room temperature for 24 h to obtain a water-soluble GAL-PEI-MUA complex.

### Synthesis of GAL-PEI-MUA-GNR (GAL-GNR) and PEI-MUA-GNR (PEI-GNR)

Freeze-dried CTAB-GNR powder (10 mg) was suspended in 10 mL of water-soluble PEI-MUA or GAL-PEI-MUA complexes. CTAB was replaced by Au–S bond when stirred at room temperature for 24 h. The solution was centrifuged at 12,000 rpm for 15 min to remove excess supernatant, and the precipitate was washed three times with distilled water. PEI-GNR or GAL-GNR powders were prepared by freeze-drying, weighed and dissolved in non-ribozyme water, and then plasma resonance effect was observed by multifunctional enzyme analyzer (Spectra Max M5e, MD, USA).

### Transmission Electron Microscopy

The GNR or GAL-GNR was suspended in distilled water, and the resuspension was placed on a copper grid. After the samples on the copper mesh were thoroughly dried in air for 30 min, the copper mesh was imaged using a transmission electron microscope (LIBRA 120, Carl Zeiss, Germany).

### Ultraviolet-Visible Analysis

The GNR, PEI-GNR, or GAL-GNR was suspended in distilled water. The surface plasmon resonance effect of nanomaterials in the wavelength range of 600 to 900 nm was examined using a multi-function microplate reader (SpectraMax M5e, MD, USA).

### Zeta Potential and Hydrodynamic Diameter Analysis

The GNR, PEI-GNR, or GAL-GNR was suspended in distilled water, and then the zeta potential and hydrodynamic diameter of the nanomaterial were measured on a Zetasizer Nano ZS.

### Nuclear Magnetic Resonance Analysis

The GAL-GNR lyophilized powder was dissolved in heavy water (99%, Sigma) to make a homogeneous suspension, and the suspension was then transferred to an NMR sample tube. The composition of GAL-GNR was determined by nuclear magnetic resonance (NMR) (Bruker, 600mhz, Germany).

### Gel Shift Assay

GAL-GNR and siBRAF were mixed according to the mass ratio (0:1, 1:1, 2:1, 3:1, 4:1, 5:1, 6:1, 7:1). After incubation at room temperature for 30 min, the DNA loading buffer was added into the mixture. All samples were added to 2% agarose gel containing 0.01% Goldview (Bioshop, USA) and then electrophoresed at 90 V for 30 min. The degree of siBRAF wrapping was visualized on the gel imaging system.

### Stability of siRNA in Serum

The mixture of GAL-GNR-siBRAF (6:1) was incubated in fresh murine serum at 37 °C for 0 h, 3 h, 6 h, 12 h, 24 h, and 48 h respectively. The naked siBRAF plus fresh murine serum group were treated under the same condition. An equal volume of 2% sodium dodecyl sulfate (SDS) was added to GAL-GNR-siBRAF solution. After incubation for 30 min at room temperature, DNA loading buffer was added, and all samples were loaded onto a 2% agarose gel (containing 0.02% Goldview (Bioshop, USA)) and electrophoresed at 90 V for 30 min. The strip brightness of the siBRAF was visualized on a gel imaging system.

### Competitive Inhibition Assay of GAL-GNR-siBRAF

2 × 10^5^ Hepa1-6 cells were cultured in 12-well microplates overnight. Then, the cells were pre-treated by Lactobionic acid for 12 h and then transfected with GAL-GNR--siBRAF (Cy3 labeled) for 30 min. The intracellular fluorescence intensity was determined by fluorescence microscope (scale bar = 200 μm).

### Liver Cancer-Targeted Delivery Ability of GAL-GNR-siBRAF

One microgram of cy3-labled siBRAF was incubated with PEI-GNR (30 μg/mL), GAL-GNR (30 μg/mL) for 20 min at room temperature, and then added to the media of Hepa1-6 cells. After incubation for 30 min, the cells were washed with 50% FBS for three times, and the fluorescence intensity in the cells was measured under a fluorescence microscope.

### Photothermal Effects

Twenty, 40, 60, 80, and 100 μg of GAL-GNR and 100 μg of GNR lyophilized powder were thoroughly dissolved in 1 mL of distilled water. The solution was added to a 24-well plate and continuously irradiated with 803 nm near-infrared laser source (2 W/cm^2^) for 15 min. The temperature of each group was recorded with an infrared thermometer every 0.5 min for the first 5 min, and then the temperature was marked every 1 min.

### MTT Assay

Two hundred microliters of cells (3.5 × 10^4^/mL) was cultured in 96-well microplates for 24 h and then cultured with different concentrations of GNR or GAL-GNR (0, 15, 30, 45, 60, 75, 90, 105, 120 μg/mL). After 24 or 48 h of incubation, 20 μL MTT was added to the culture system and incubated for an additional 4 h. The purple crystals were dissolved in 150 μL DMSO, followed by spectrophotometric analysis at 490 nm using a reference of 650 nm in a microplate reader (SpectraMax M5e, MD, USA).

### Calcein-AM and PI Staining Test

Hepa1-6 cells (1.5 × 10^5^) were kept overnight in a 12-well plate and treated with PBS, laser, GAL-GNR-siBRAF (30 μg/mL:1 μg), GAL-GNR-siGL2 (30 μg/mL:1 μg) + laser or GAL-GNR-siBRAF (30 μg/mL:1 μg) + laser for 4 h, and then irradiated by near infrared light (808 nm, 2 W/cm^2^) for 15 min. The cytotoxicity of GAL-GNR was detected by calcein-AM (live cells) and PI (dead cells) staining experiments. Green fluorescence of calcein-AM and red fluorescence of PI were photographed under fluorescence microscopy.

### Real-Time Quantitative PCR

Total cellular RNA was extracted using Trizol (Trizol reagent, Invitrogen) and used as a template for cDNA synthesis. Q-PCR was conducted using gene-specific forward and reverse primers (0.5 μL × 10 μM each) in the Stratagene Mx3000P qRT-PCR system (Agilent Technologies, Lexington, MA, USA). SYBR green PCR MasterMix (Life Technologies) was used according to the manufacturer’s scheme. Housekeeping gene β-actin was used as an internal reference. The primer sequences were β-actin: 5′AGGGAAATCGTGCGTGACATCAAA-3′ (forward) and 5′ACTCATCGTACTCCTGCTTGCTGA-3′ (reverse); BRAF: 5′-CAATTGGCTGGGACACGGACAT-3′ (forward) and 5′-TTGACAACGGAAACCCTGGAAAAG-3′ (reverse). The difference of gene expression was calculated and displayed by 2^−∆∆Ct^ method.

### Western Blot

The total protein of Hepa1-6 cells were obtained by RIPA buffer (Cell signaling, Pickering, Ontario, Canada) and quantified by bicinchoninic acid (BCA). Protein was separated in 12% SDS-PAGE and transferred to Polyvinylidene fluoride (PVDF). The housekeeping gene β-actin was used as an internal reference to detect BRAF protein expression. The protein band and internal reference of the target protein were detected by ECL chemiluminescence.

### Scratch Test

The transfected Hepa1-6(1.7 × 10^5^/mL)cells were seeded into 12-well plates overnight. Cells that completely adhered to the plate were scratched with a 10-μL pipette tip. The cells were washed twice with PBS and replaced with medium containing 2% FBS. The average of the wound line was observed under a microscope after 0, 24, and 48 h. Each scratch test was performed in triplicate.

### Transwell Assay for Migration and Invasion

Eight-micrometer transwell chambers (Corning Life Science) were used to assess cell migration and invasion. Hepa1-6 suspended in 200 μL of serum-free medium were added to the upper chambers at a density of 1.7 × 10^5^ mL cells/well, and then 500 μL of medium containing 12% FBS was added to the lower chamber. After incubation for 24–48 h, the cells were fixed with a 4% paraformaldehyde solution for 30 min, and then the cells were stained with a 1% crystal violet solution for 30 min. Finally, the cells were photographed and counted under an inverted microscope. For the cell invasion assay, cell invasion experiments require ECM gel coating, and the remaining steps are consistent.

### Statistics

All experiments were performed in triplicate. Data were expressed as mean ± SD and Student’s *t* test (two-tailed) to determine the difference between the two methods. A one-way ANOVA test was used for comparison of multiple groups. Data were considered statistically significant at *p* < 0.05 (*p < 0.05, **p < 0.01, ***p < 0.001).

## Results

### Synthesis and Characterization of Nanocarrier GAL-GNR-siBRAF

**Scheme 1:**
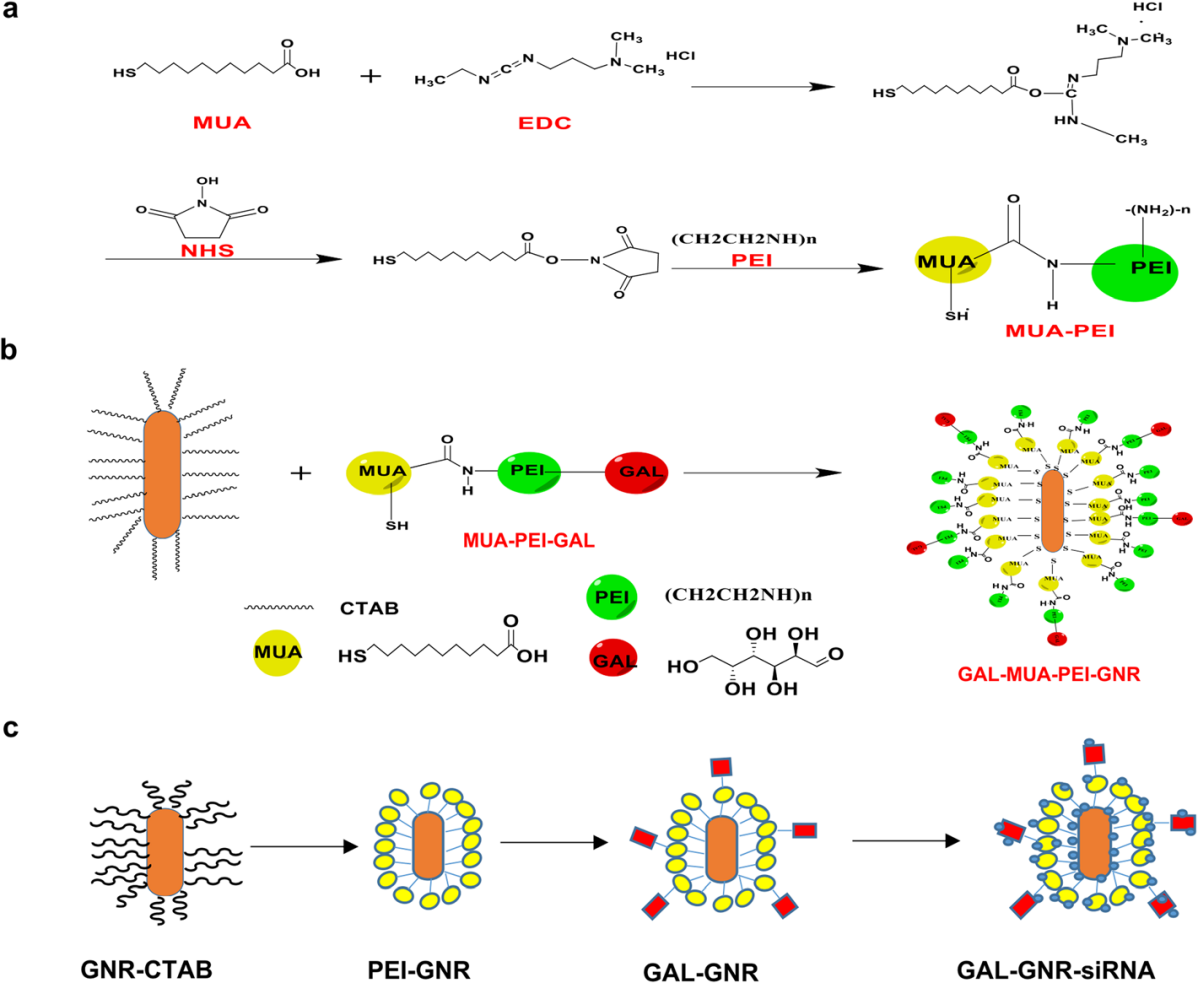
GAL-GNR synthetic procedure. **a** The synthetic process of MUA-PEI. **b** Activation of d-galactose and chemical reaction with MUA-PEI. **c** The final synthetic product of GAL-GNR

**Fig. 1:**
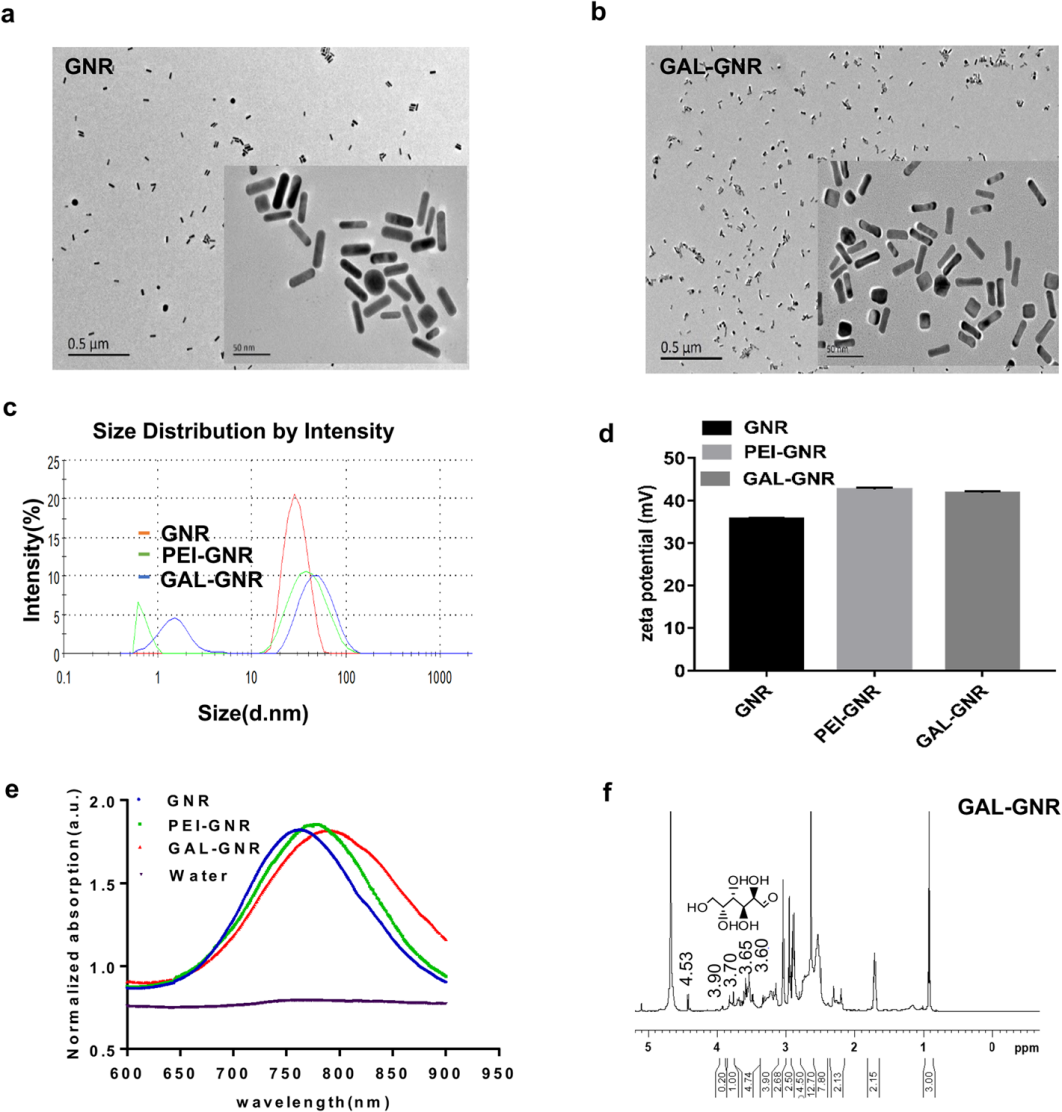
Characterization of GNR and GAL-GNR. **a** TEM micrograph of GNR (scale = 0.5 μm/50 nm). **b** TEM micrograph of GAL-GNR (scale = 0.5 μm /50 nm). **c** Particle size analysis of different modified GNR. **d** Zeta potential analysis of different modified GNR (GAL-GNR). **e** Normalized UV–Vis absorption spectra of different modified GNR and water. **f** NMR absorbance spectra of GAL-GNR

We designed a novel nanocarrier GAL-GNR that is capable of delivering small interfering RNA (siRNA) to the liver cells and maintaining the photothermal effect of the gold nanorods simultaneously. The synthesis procedure of GAL-GNR is shown in Scheme 1. This liver-targeted system contains three functional components. Firstly, the basic part of GAL-GNR is a GNR skeleton, which is about 30 nm in length and 10 nm in diameter, as shown in the TEM image (Fig. 1a) and chemically conjugated GNR (GAL-GNR) showed no significant dimensional change and still possessed well dispersibility (Fig. 1b). The data of the particle size showed that the average size of GNR was 30.23 nm which was consistent with the results of electron microscopy, and the size of GAL-GNR (50 nm) and PEI-GNR (42.35 nm) was larger than GNR since the conjugation of GAL and PEI increased the hydration between particles (Fig. 1c). Secondly, biologically toxic CTAB on the surface of GNR was replaced by positively charged MUA-PEI which can be loaded with negatively charged siRNA. The zeta potential measurement showed that the surface charge of GNR increased from 35.6 to 42.7 mV or 41.8 mV when the GNR were modified with PEI or GAL-PEI respectively, indicating that GAL-GNR possessed strong ability to bind siRNA (Fig. 1d). Thirdly, we applied GAL as a guide molecule to conjugate GNR nanocarriers, which can be used for specific homing of hepatocellular carcinoma. UV-Vis absorption spectroscopy was used to detect the structure of modified GNR preliminarily. The initial absorption spectrum wavelength of unmodified GNR was 763 nm; a shift of 7 nm in wavelength was observed initially with the MUA-PEI modification, and another shift of 8 nm was observed at the end of the synthesis, when the modification of GNR with GAL successfully (Fig. 1e). In order to confirm GAL in the nano-system, NMR image was used to analyze the chemical groups. The results showed that the H signal of galactose was only found in the GAL-GNR spectrum on the NMR hydrogen spectrum, which was δ: 3.60, 3.65, 3.70, 3.78, 3.90, 4.53. NMR spectra confirmed the chemical structure of GAL, in which GAL was successfully conjugated to the GNR surface (Fig. 1f).

### siRNA Encapsulation Ability and Stability of GAL-GNR

**Fig. 2:**
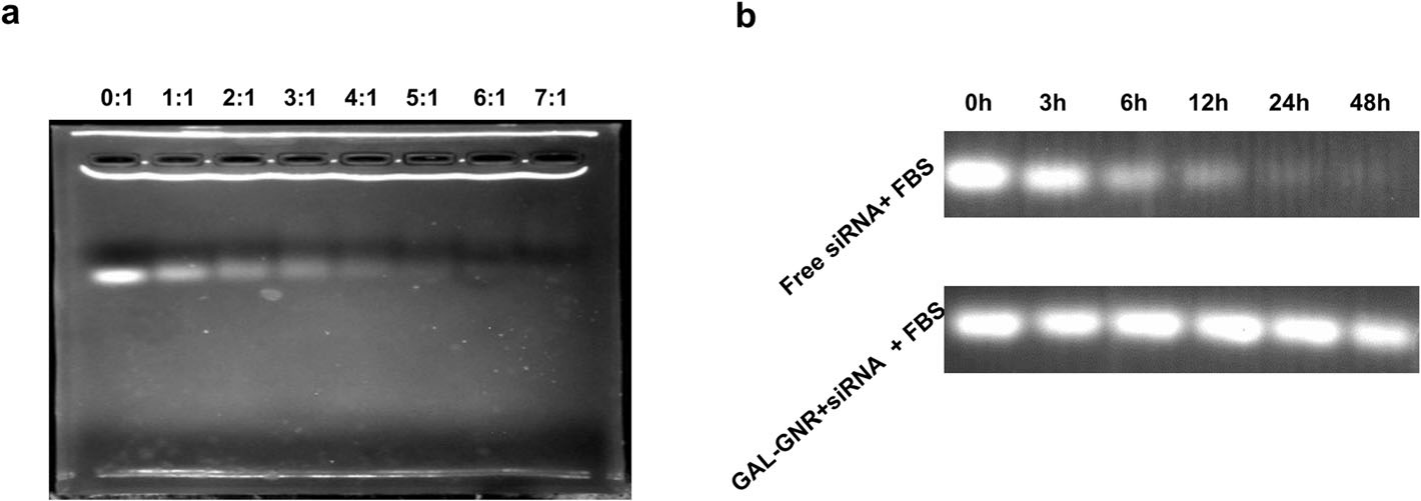
Gel shift assays. **a** siRNA-loading capacity of GAL-GNR. One microgram of siRNA was mixed in equal volumes with different mass ratios of GAL-GNR. After incubation for 30 min, a gel migration assay was used to assess the siRNA loading capacity of GAL-GNR. **b** Stability of siRNA in mouse serum. One microgram of BARF siRNA was inoculated with the GAL-GNR at the mass ratio of 1:6 and was subsequently added to fresh murine serum. After reaction of 0, 3, 6, 12, 24, and 48 h at 37 °C, siRNA from GAL-GNR-siBARF was extracted by 2% SDS and were visualized with Ethidium Bromide

The GAL-GNR nano-skeleton was modified by positively charged PEI, which can be combined with negatively charged siRNA through electrostatic interaction. We used gel shift assay to evaluate the siRNA binding ability of GAL-GNR. The free siRNA can move along the passageway of gel while the bound siRNA is slowed down or totally stopped. In addition, the bound siRNA will no longer bind effectively to bromide, and thus, the fluorescence intensity will decrease accordingly. The gel electrophoresis result showed that the optimal ratio for saturating GAL-GNR with siBRAF is 6:1 (w/w) (Fig. 2a).

Naked siRNA is very degradable, especially in vivo. An important function of the delivery system is to protect siRNA from degradation. Thus, we tested the protective effect of nanocarrier GAL-GNR on siRNA in fresh serum consequently. Compared with the naked siRNA, the bound siRNA was stored in serum for longer periods of time. As shown in Fig. 2b, naked siRNA could exist in serum for up to 12 h while the bound siRNA was still abundant after 48 h. The result demonstrated that GAL-GNR can effectively prevent siRNA from being degraded in serum, ensuring targeted delivery in vivo.

### Cytotoxicity of Nanomaterials

**Fig. 3:**
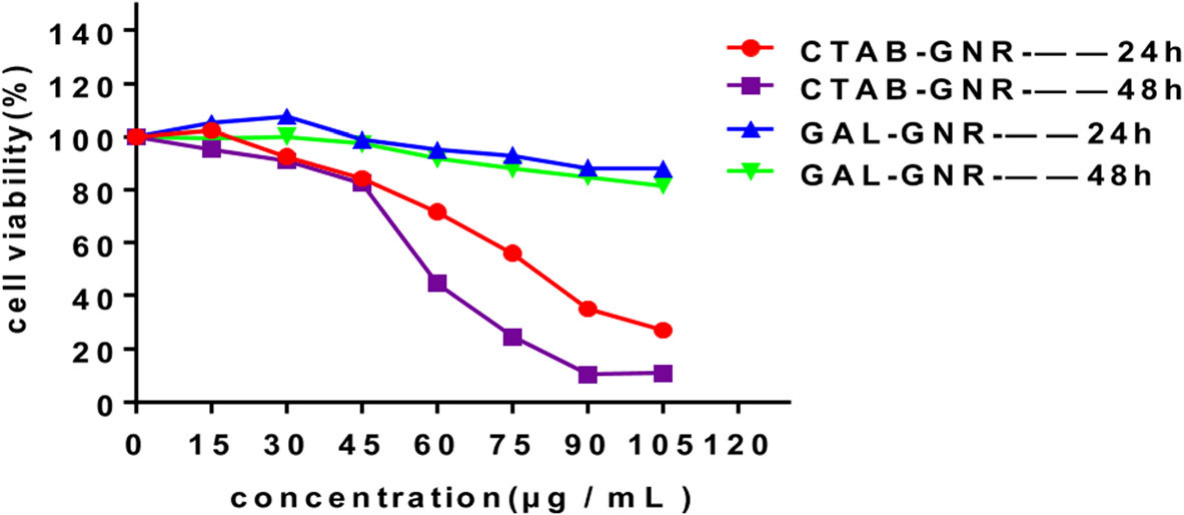
Toxicity of GAL-GNR. Hepa1-6 cells were treated with GNR (CTAB-GNR) or GAL-GNR for 24 or 48 h at the indicated concentrations. Cell viability was measured by MTT assay (*n* = 5)

Low biological toxicity is another important property of nanomaterials, which enables them to be used in cancer treatment. To evaluate the biocompatibility of this new GAL-GNR nanostructure, we tested its cytotoxicity. As shown in Fig. 3, dead cells of Hepa1-6 in the unmodified GNR-treated group was observed initially at a low concentration of 30 μg/mL. After incubation with 75 μg/mL GNR for 24 h, the cell death rate was up to 56.09%, and the mortality increased to 75.5% after incubation for 48 h. On the contrary, GAL modification reduced cytotoxicity significantly. After incubating GAL-GNR for 48 h, more than 80% cells were still alive even at high concentration (105 μg/mL). These data suggested that the nanocarrier GAL-GNR had high biocompatibility as compare with GNR, ensuring safety of application.

### Hepatoma Cells-Targeted Delivery of siRNA and Gene Silencing of BRAF In Vitro

**Fig. 4:**
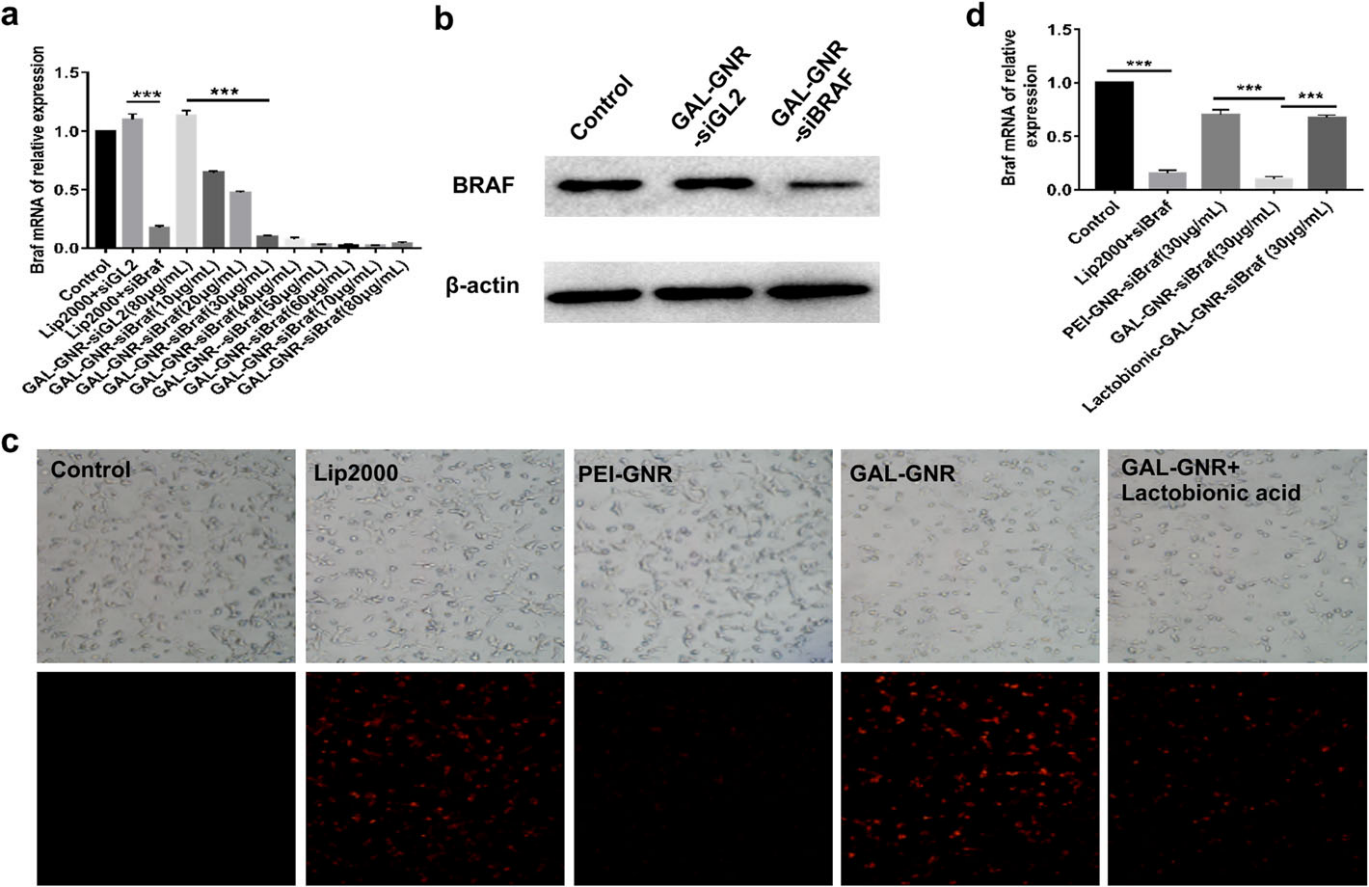
Hepatoma cell-targeted delivery of siRNA and gene silencing of BRAF in vitro. **a** The mRNA of BRAF expression after GAL-GNR-siBRAF transfection. Hepa1-6 cells were transfected with various concentrations of GA-GNR carrying the same amount BRAF siRNA (1 μg) for 24 h. The expression of BRAF mRNA was determined by qPCR. The error bar represents the standard deviation of three experiments (***p < 0.001). **b** The protein expression of BRAF in Hepa1-6 after GAL-GNR-siBRAF transfection. Hepa1-6 cells were transfected with GAL-GNR-siBRAF, GAL-GNR-siGL2, or PBS for 48 h, the levels of BRAF and β-actin were determined by Western blot. **c** and **d** Liver cancer-targeted delivery ability of GAL-GNR-siBRAF. One microgram of cy3-labled siBRAF was incubated with lip2000, PEI-GNR (30 μg/mL), GAL-GNR (30 μg/mL) for 20 min at room temperature and then added to the media of Hepa1-6 cells. For competitive inhibition assay, Hepa1-6 cells were pre-treated with Lactobionic acid overnight. The intracellular fluorescence intensity was observed under the fluorescence microscope (scale bar = 200 μm), and the mRNA expressions of BRAF were quantified by q-PCR. The error bar represents the standard deviation of three experiments (*p < 0.05)

The capacity to deliver siRNA into cancer cells is one of the most important function of nanomaterials. To investigate the potential of GAL-GNR in siRNA delivery, we detected the efficacy of gene silencing using siBRAF loaded by GAL-GNR in Hepa1-6 cells. Compared with the blank group, the expression of BRAF in GAL-GNR-siGL2 (negative control) transfected cells has no change while the cells transfected with GAL-GNR-siBRAF had a significant decrease (Fig. 4a). Moreover, the silencing effect was dose-dependent, which is correlated with the increase of GAL-GNR concentration. The qPCR results demonstrated that the ideal transfected concentration of GAL-GNR-siBRAF was 30 μg/mL, and the silencing efficiency can reach to 90.29% which was similar to the group of lipofectamine 2000 (positive control). The result of western blot was consistent with qPCR, and the protein expression of BRAF was downregulated after 48 h transfection (Fig. 4b).

Lactobionic acid is similar to GAL in structure which can be competitively combined with the asialoglycoprotein receptor (ASGPR) that is expressed on the surface of hepatocellular carcinoma cell membrane [35]. To confirm the specific targeting efficiency of GAL-GNR, Hepa1-6 cells were pre-incubated with lactobionic acid and then transfected with GAL-GNR-siBRAF. Untreated cells were used as blank control, and classical transfection reagent lipofectamine 2000 was used as positive control. As shown in Fig. 4c, the red fluorescence intensity of GAL-GNR-transfected cells was similar to the positive control group, while the fluorescence intensity of PEI-GNR-transfected cells is significantly reduced. On the other hand, cells transfected with GAL-GNR-siBRAF alone were observed stronger red fluorescence of Cy3 than Hepa1-6 cells pretreated with lactobionic acid (Fig. 4c), and the result of qPCR was consistent with that of red fluorescence results (Fig. 4d). These results further illustrated that GAL effectively promoted the endocytosis of siRNA by Hepa1-6 cells and can be applied as targeted moiety of HCC.

### The Impact of GAL-GNR-siBRAF on Proliferation, Migration, and Invasion of Hepa1-6 cells

**Fig. 5:**
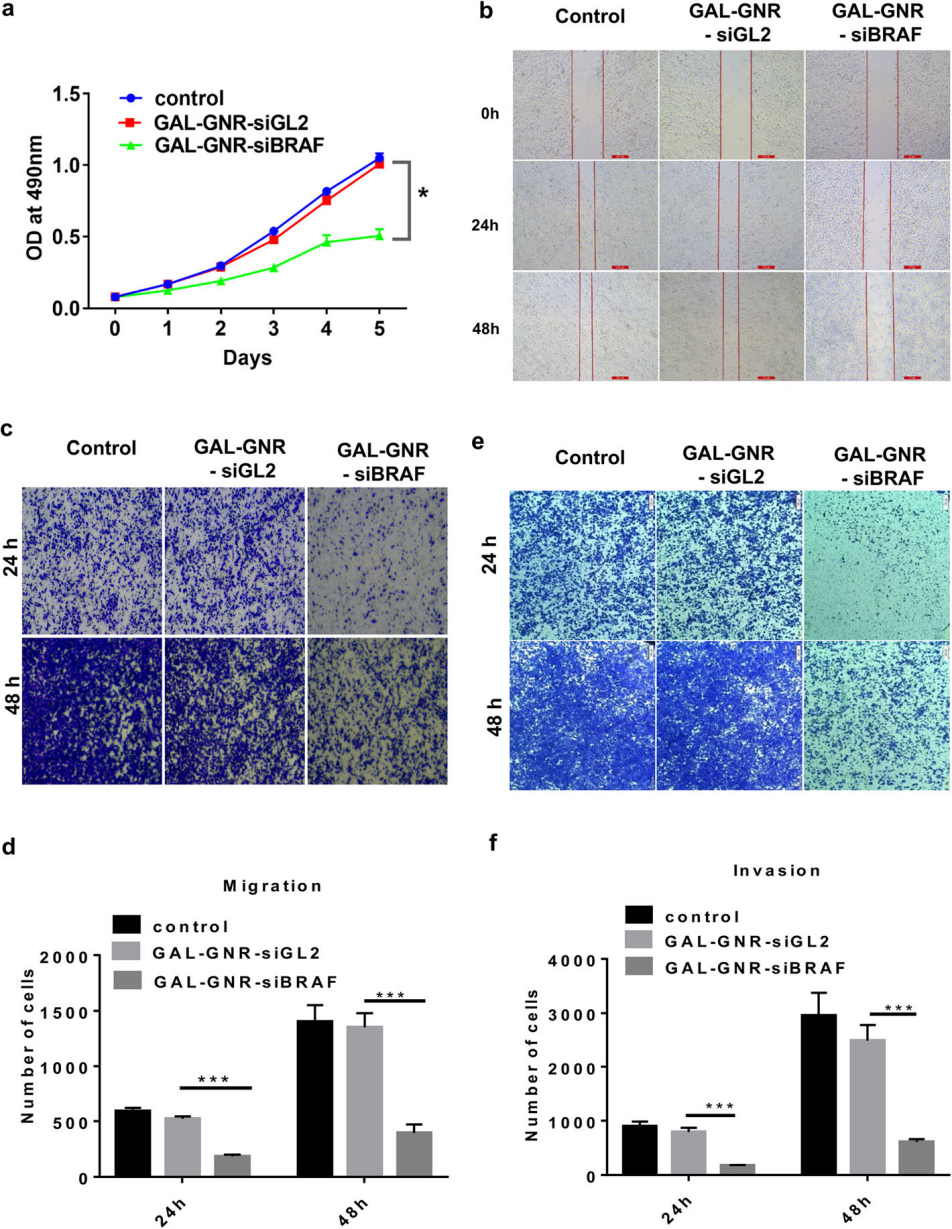
The impact of silencing BRAF gene on Hepa1-6 cells by GAL-GNR-siBRAF. **a** Proliferation of Hepa1-6 Cells. Hepa1-6 cells were transfected with GAL-GNR-siBRAF, GAL-GNR-siGL2, or PBS, and the cell proliferation was measured by MTT assay (*n* = 5). The error bar represents the standard deviation of three experiments (*p < 0.05). **b** Scratch test of Hepa1-6 cells. Hepa1-6 cells were transfected for 24 h as described above and then scraped with a 10 μL tip. The cell scratch images were taken at different time points (scale bar = 200 μm). **c** and **e** Migration and invasion of Hepa1-6 cells. Hepa1-6 cells were transfected for 24 h as described above. Cell migration and invasion ability was determined by transwell assay, and imagines were taken at different time points (scale bar = 200 μm). **d** and **f** Columnar statistical analysis of cell migration and invasive capacity (***p < 0.001)

BRAF plays an important role in MAPK pathway, which is responsible for regulating cell proliferation, migration, and invasion [36]. To assess the impact of GAL-GNR-siBRAF on hepatocellular carcinoma cell, we analyzed the cell proliferation by MTT assays first. As shown in Fig. 5a, the proliferation of GAL-GNR-siBRAF-transfected hepa1-6 cells was significantly decreased compared with the GAL-GNR-siGL2 (negative control) or PBS-treated cells. Next, we investigated the effect of GAL-GNR-siBRAF on cell migration by scratch and transwell assays. Compared with negative control cells, the migration ability of Hepa1-6 cells that transfected with GAL-GNR-siBRAF was evidently reduced (Fig. 5b–d).

To further investigate the potential of GAL-GNR-siBRAF on the invasion, transwell assays were performed. The results showed that hepa1-6 cells transfected with GAL-GNR-siBRAF haven significantly decreased the invasion of Hepal-6, as compared with GAL-GNR-siGL2 or PBS (Fig. 5e and f). The above results implied that knockdown of BRAF gene using GAL-GNR-siBRAF complexes can effectively reduce migration and invasion of hepatocellular carcinoma cells.

### Combination Treatment with Photothermal Effect and Gene Silencing of GAL-GNR-siBRAF

**Fig. 6:**
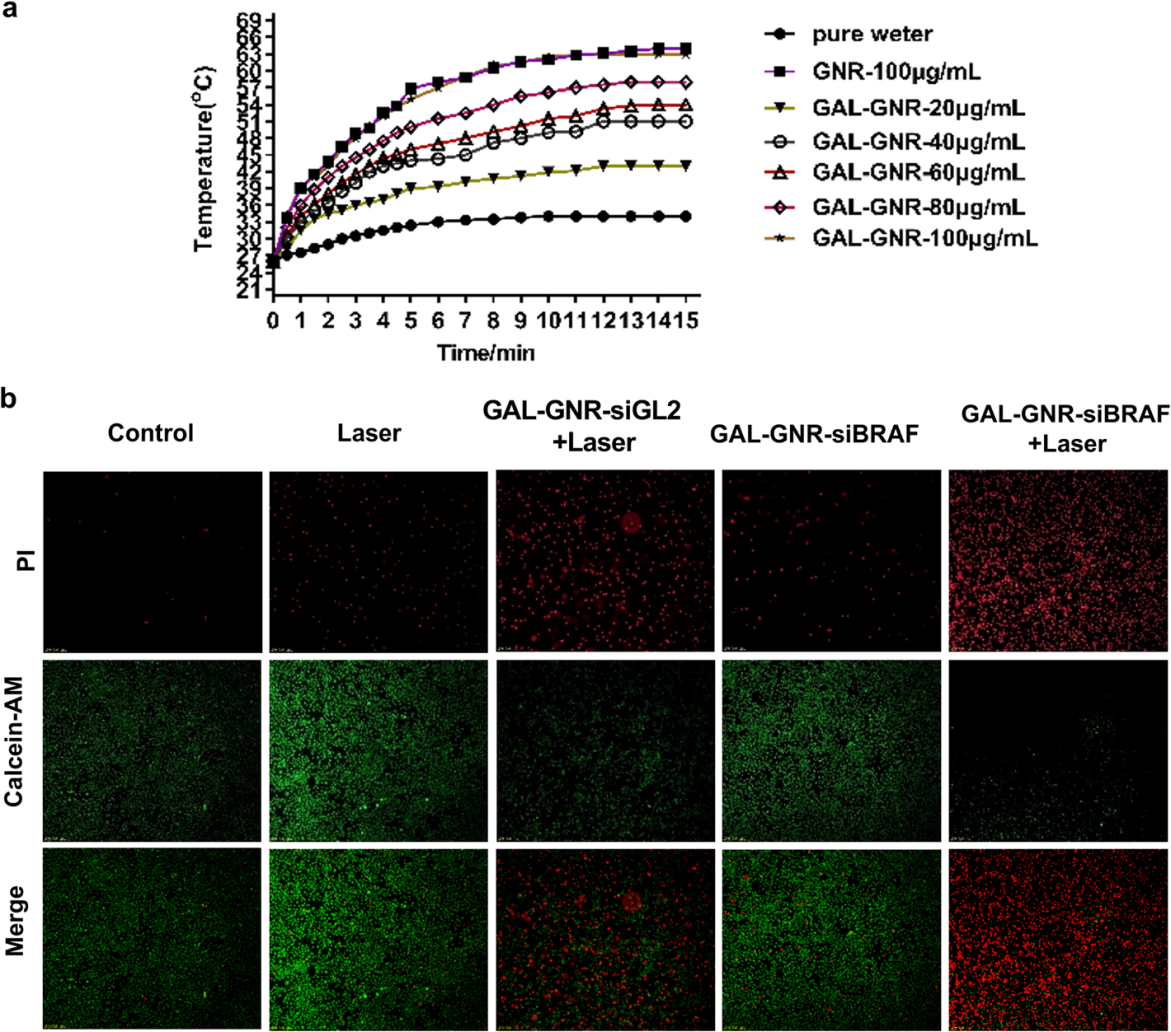
Photothermal effect induced by GAL-GNR and combination treatment of photothermal effect and gene silencing on liver cancer cells. **a** 803 nm near-infrared laser source (2 W/cm^2^) was used to irradiate the solution of GNR, GAL-GNR, or distilled water (blank control) for 15 min. The temperature was detected by infrared thermometer at different time point. **b** Hepa1-6 cells were treated with PBS, laser, GAL-GNR-siBRAF, GAL-GNR-siGL2 + Laser, or GAL-GNR-siBRAF + Laser, respectively. Green fluorescence of calcein AM (live cells) and red fluorescence of PI (dead cells) were observed under fluorescence microscope

Our previous studies have demonstrated that gold nanomaterials can generate thermal energy under near infrared radiation [37]; this phenomenon is called photothermal effect due to LSPR properties of the GNR. Our results showed that, with the increase of GAL-GNR concentration, the heat production capacity of GAL-GNR was enhanced. When the concentration of GAL-GNR is 100 μg/mL, the temperature quickly rises from 24 to over 60 °C. In addition, there was no significant difference in heat production capacity between GNR and GAL-GNR (Fig. 6a).

The novel nano-system GAL-GNR-siBRAF possesses three individual characteristics: specific targeting of liver cancer, siRNA-based gene silencing of BRAF, and GNR-offered photothermal effects. Thus, we further study the synergistic therapeutic effect of GAL-GNR-siBRAF in anti-tumor treatment. Hepa1-6 cells without GAL-GNR kept higher viability even under near infrared radiation, suggesting laser itself had no significant effect on tumor cells. Once the laser irradiation was carried out on the basis of GAL-GNR-siGL2 treatment, the photothermal effect showed powerful killing ability on Hepa1-6 cells. Although BRAF gene silencing alone induced cell death, the effect was not conclusive. The treatment of cells with the nano-material GAL-GNR-siGL2 plus laser irradiation resulted in much stronger cell-killing ability than BRAF gene silencing individual (Fig. 6b). These data indicates that the BRAF gene silencing and photothermal effect can synergistically increase the potential of killing tumor cells.

## Discussion

Difficulties in tumor-targeted delivery are major obstacles in the clinical treatment of tumors. This study demonstrated for the first time that we have developed a novel multifunctional nanocarrier GAL-GNR-siBRAF, and it was provided with many advantages. The modified GAL-GNR not only possessed good biocompatibility but also maintained the LSPR phenomena of GNR skeleton and still has excellent photothermal conversion ability. GNR modified with GAL can home hepatocellular carcinoma cells through ASGPR, which has great potential in targeted molecular therapy of hepatocellular carcinoma. Further studies showed that BRAF gene silencing inhibited the proliferation, migration, and invasion of hepatoma cells. It is worth noting that GAL-GNR-siBRAF shows a stronger cell killing ability in the combination of photothermal effect and gene silencing of BRAF.

RNA interference (RNAi) has attracted much attention due to its crucial role in gene expression and regulation. Using siRNA to knock down sequence-specific genes in cancer cells for gene therapy is an effective and specific targeted gene therapy, and which has become a new therapeutic tool for many diseases including cancer [29, 31]. However, low stability in serum and poor cell uptake limit siRNA in clinical application [38]. On the other hand, siRNA possess negative charge that prevents it from binding negatively charged cell membranes, and siRNA itself is unlikely to be delivered directly into cells [32, 39]. In addition, there are problems associated with low transfection efficiency and poor cell internalization in siRNA therapy [40]. These barriers impede the delivery of siRNA to target cells. To enhance siRNA stability and to strengthen gene silencing efficacy, we synthesized a novel nanocarrier, GAL-GNR-siBRAF, which includes three components (Scheme 1). This nanocarrier has low molecular size (about 30-nm longitude and 10-nm diameter) and good dispersibility (Fig. 1). Additionally, GAL-GNR-siBRAF could effectively load large amount of siRNA at low concentration (Fig. 2a) and protect siRNA degradation from RNase in serum; the siRNA was evidenced by stability for at least two days at 37 °C in the presence of fresh murine serum (Fig. 2b).

Good biocompatibility is essential for nanomaterials used in the field of biotherapy. CTAB is an essential active reagent in the synthesis of gold nanorods although it has apparent cytotoxicity that limits its biological application [41]. In order to reduce the cytotoxicity of the material, we manipulated the GNR by external modification of positive charge PEI and targeting adaptor GAL (d-galactose). Our results showed that, compared with unfunctionalized GNR, GAL-modified GNR has a better biocompatibility. The cell viabilities maintained over 80% even at very high concentrations (105 μg/mL) of GAL-GNR for 48 h (Fig. 3). In addition, the small molecular size and cylindrical shape of the nanorod facilitate its penetration through the cell membrane into the cell [37, 38, 42]. This phenomenon is particularly evident in targeted therapy of cancer cells. Nano-construct modified with the galactose-targeting moieties resulted in high accumulation of GAL-GNR-siBRAF in tumor cells, inducing effective downregulation of BRAF gene expression (Fig. 4). Moreover, according to the competitive inhibition experiments, we found that GAL-GNR entered cells mainly through ASGPR surface receptors of Hepa1-6 cells (Fig. 4c).

It was reported that HCC with high expression of BRAF and RAF1 tends to have rapid proliferation and growth [41, 43, 44]. B-Raf and Raf1 mainly act on the downstream of ERK/MAPK pathway, regulating nuclear factors through cascade amplification. Activation of this pathway accelerates the proliferation and differentiation of HCC cells abnormally [41, 44, 45]. RAF gene can also promote the expression of matrix metalloproteinases (MMPs), which can change the adhesion of tumor cells, degrade extracellular matrix (ECM) and basement membrane, and promote the invasion and metastasis of tumors [44, 45]. BRAF kinase regulates the RAS-RAF-MEK-ERK pathway, which promotes tumor cell proliferation, invasion and metastasis, and allows cell death through apoptosis [5, 46, 47]. In previous work, we found that BRAF gene was highly expressed in Hepa1-6 cells (data were not shown). It is speculated that the knock down of BRAF expression in Hepa1-6 will block the RAS-RAF-MEK-ERK pathway and lead biological changes of Hepa1-6. To verify our hypothesis, we transfected hepatocellular carcinoma cells with GAL-GNR-siBRAF and found that it can restrain the cell proliferation, migration, and invasion significantly (Fig. 5), providing a new strategy for clinical treatment of hepatocellular carcinoma.

Another important advantage of GNR is that it possesses local surface plasmon resonance (LSPR), which can convert absorbed light energy into heat energy under near infrared light irradiation, thereby killing and destroying cells [42]. Then, we explored the light-to-heat conversion ability of GAL-GNR, and the results showed that the modified GAL-GNR can induce heat energy effectively under the irradiation of near infrared light (Fig. 6a). Next, we further investigated the synergistic effects of GAL-GNR-siBRAF. Whereas BRAF gene silencing showed limited cytotoxicity, the treatment of GAL-GNR-siGL2 + laser had a much stronger killing ability on tumor cells. At the same time, the combination of photothermal hyperthermia and BRAF gene silencing could cause more than 85% cell death (Fig. 6b) which indicates that the synergy of GAL-GNR-siBRAF and photothermal effects could be an ideal strategy to inhibit liver cancer.

## Conclusion

This study showed that GAL-GNR-siBRAF overcome the obstacle of siRNA degradation, effectively increased the stability of siRNA in serum in vitro. In addition, GAL-GNR-siBRAF can target deliver siRNA to hepatocellular carcinoma cells and knockdown the expression of BRAF and inhibit the cell proliferation, invasion, and migration significantly. More importantly, GAL-GNR-siBRAF, as a new multifunctional nanocarrier, can greatly enhance the ability of killing tumor cells when combined with near-infrared light. In conclusion, GAL-GNR-siBRAF has great potential in treatment of hepatocellular carcinoma and provides new ideas for clinical application of liver cancer.

## Data Availability

All data generated and materials used in this study are included in the manuscript and corresponding additional files.

## Abbreviations

GAL: d-galactose
GNR: Golden nanorods
siBRAF: Small interfering RNA specific for BRAF
HCC: Hepatocellular carcinoma
ASGPR: Asialoglycoprotein receptor
TACE: Transcatheter arterial chemoembolization
LSPR: Localized surface plasmon resonance
CTAB: cetyltrimethylammonium bromide
MUA: Mercaptoundecanoic acid
EDC: 1-ethyl-3-[3-dimethylaminopropyl] carbodiimide hydrochloride
NHS: N-hydroxysuccinimide
PEI: Polyethyleneimine
TEM: Transmission electron microscopy
NMR: Nuclear magnetic resonance
FBS: Fetal bovine serum
DMSO: Dimethyl Sulfoxide
BCA: Bicinchoninic acid
PVDF: Polyvinylidene fluoride
